# An immunogenic cell death-associated classification predictions are important for breast invasive carcinoma prognosis and immunotherapy

**DOI:** 10.3389/fgene.2022.1010787

**Published:** 2022-10-20

**Authors:** Xinghe Liao, Hui Liu, Zhe Zhang, Jing Zhang, Chenyue Zhang, Weiwei Zhao

**Affiliations:** ^1^ Department of Integrated Therapy, Fudan University Shanghai Cancer Center, Shanghai, China; ^2^ Department of Oncology, Shanghai Medical College, Fudan University, Shanghai, China; ^3^ Department of Neurology, Renmin Hospital of Wuhan University, Wuhan, China

**Keywords:** immunogenic cell death, breast invasive carcinoma, prognosis, tumor immune microenvironment, biomarker

## Abstract

As a type of regulated cell death (RCD), immunogenic cell death (ICD) can initiate the adaptive immune responses. Numerous reports highlight the capacity of ICD to alter the tumor immune microenvironment by releasing Damage-Associated Molecular Patterns (DAMP) or danger signals to boost the efficacy of immunotherapy. Therefore, identification of the ICD-associated biomarkers is crucial for the prediction of ICD-induced immune responses. In this report, the consensus clustering technique was used to identify two subcategories (subtypes) linked to ICD. In comparison to the ICD-low subcategory, the ICD-high subcategory showed longer survival and more immune cell infiltration. Then, a novel ICD-associated prognostic model was developed and validated for predicting the survival of patients with breast invasive carcinomas (BRCA) and is linked to the tumor immune microenvironment. To conclude, a novel ICD-based BRCA classification scheme was designed. For individuals with BRCA, this categorization will be crucial for directing the assessment of prognosis and treatment.

## Introduction

As a type of regulated cell death (RCD), immunogenic cell death (ICD) can trigger the adaptive immune responses (Garg et al., 2015; Galluzzi et al., 2020). Several researches published in the last few years have introduced the notion of ICD. Tumor cells can induce ICD by expos to calreticulin (CRT) and releasing high mobility group protein B1 (HMGB1), this promotes the recruitment of antigen presenting cells (APCs) and tumor infiltration of T cells, which contribute to the activation of antitumor immune responses (Sun et al., 2020; Jin and Wang, 2021). Cancer immunotherapy refers to the utilization of the body’s immune system to stimulate antitumor immune responses. Numerous trials have explored the antitumor role of ICD *via* the activation of immune responses (Ahmed and Tait, 2020). The ICD is important in combating cancer because of its ability to elicit antitumor immune responses and thereby enhance the therapeutic efficacy of chemotherapeutic and radiotherapeutic agents (Troitskaya et al., 2022). Although ICDs have been employed in a variety of preclinical models, there have been insufficient data on their clinical application (Fucikova et al., 2020). Therefore, further research on ICD-linked studies in patients could be performed in a clinical setting. It is crucial to detect biomarkers that can categorize the patients based on their response to ICD immunotherapy.

The incidence of breast cancer in women will ranking first among all types of cancers with a global occurrence (SUNG et al., 2021). Since breast cancer has a lower mutational load and is immunogenic, it is considered a “cold” tumor. However, recent research has found that both the Human Epidermal growth factor Receptor 2 (HER-2)-Positive and the Triple Negative (TN)-breast cancers have a higher expression level of tumor-infiltrating lymphocytes (TILs) and programmed death ligand 1 (PD-L1) (Dieci et al., 2021). The immune checkpoint inhibitors (ICIs) that target the PD-L1 and programmed death-1 (PD-1) could promote immunogenicity and modify the tumor microenvironment (TME) to enhance the therapeutic effects. Efforts to identify precise biomarkers to predict immunotherapies in BRCA would become more prominent as a result of the ongoing development of cancer immunotherapies, improved knowledge of T cell responses to targeted immune checkpoint medications, and the success of the clinical investigation of drugs that block these ICI molecules.

For the purpose of predicting the prognosis, immunological milieu, and response to immunotherapy in BRCA, a novel ICD risk model was developed in this study with the goal to identify ICD-associated biomarkers. In the future, hopefully, it will be useful for clinical decision-making.

## Experimental procedures

### Datasets used in the study

The Cancer Genome Atlas (TCGA) is a publicly funded project to catalog and discover major oncogenic genomic alterations. Clinicopathological factors and the RNA-sequencing transcriptome data regarding BRCA (993 tumors and 107 normal) were derived from the NCI-TCGA dataset (https://portal.gdc.cancer.gov/).

### GeneMANIA

GeneMANIA (http://www.genemania.org) (Vlasblom et al., 2015) is a web site for generating hypotheses about gene function, analyzing gene lists and prioritizing genes for functional assays, and was employed for predicting the gene and protein interactions, pathways, and functions of ICD-associated mRNAs as well as their related interactors.

### Designing a new protein-protein interaction network

Herein, a new protein-protein interaction (PPI) network was investigated using the online software of Search Tool for Retrieval of Interacting Genes (STRING) (https://string-db.org/) (Szklarczyk et al., 2019). Differential expression of the ICD-associated mRNAs and their likely interactions were determined and integrated using the PPI network analysis.

### Consensus clustering

In this report, consensus clustering was carried out with a ConsensusClusterPlus tool in the R software for identifying the molecular subtypes associated with ICD. Subsequently, for ensuring the accuracy of the derived results, the optimal number of clusters was assessed for k = 2–10, and the complete process was repeated 1000 times. The cluster maps were created using the Pheatmap tool in R.

### MethSurv

The researchers investigated the prognostic significance of the single CpG methylation status of ICD-associated mRNAs in the BRCA patients using MethSurv (https://biit.cs.ut.ee/methsurv/). It is a web platform that helps in survival analysis based on the CpG methylation pattern.

### Identifying the differentially expressed genes (DEGs)

Here, the t.test function of the R software was used for assessing the significance of each mRNA across different groups. The p. adjust function was used to calculate a significant FDR for each gene to ultimately obtain the differential information for each gene. The following screening criteria were followed for determining the differential expression of the mRNAs: adjusted *p*-value<0.05 and | fold change|>1.5.

### Functional enrichment analysis of the data

Herein, the Kyoto Encyclopedia of Genes and Genomes (KEGG) and the Gene Ontology (GO) analyses were used for comparing the biological effects and differential signal pathways across the ICD-high and -low cohorts. The KEGG rest API tool (https://www.kegg.jp/kegg/rest/keggapi.html) was used in this study. The most recent gene annotation of the KEGG pathway was taken for enrichment analysis using the clusterprofiler tool from the R-package (ver. 3.14.3) for deriving the gene set enrichment analysis (GSEA) results. The GO annotation of all genes from the R package org. Hs. eg.db (ver. 3.14.3) was utilized to map the genes into a background set, with the R package clusterProfiler as the background for the purpose of deriving the results of the functional enrichment analysis of the gene sets. A *p*-value<0.05 and an FDR<0.1 were regarded to be statistically significant, with the maximal gene set size of 5000 and minimal gene set size of 5.

### Gene set enrichment analysis of the data

The enrichment scores for Gene set enrichment analysis (GSEA) were acquired from the GSEA dataset (http://software.broadinstitute.org/gsea/index.jsp). The ICD-low and -high cohorts were combined, websites were retrieved using GSEA software (ver. 3.0), and the molecular signature database (http://www.gsea-msigdb.org/gsea/downloads.jsp) was used. The C2 cp. kegg. v7.4. symbols were further acquired. Furthermore, the GMT subset was used for assessing the molecular mechanisms and relevant pathways, based on phenotypic grouping and gene expression profiles. We set the maximal gene set size of 5000, 1000 resampling, and a minimal gene set size of 5. Values with a *p* < 0.05 and FDR<0.1 denoted a statistically significant difference.

### Characterization of immune landscape between 2 ICD subgroups

An immune infiltrating cell score was generated for each sample based on the expression profiles using the R package IOBR utilizing Cibersort and Estimate techniques. IOBR is a computational tool that is generally used for immune-tumor biology investigations.

### Somatic mutation analysis

The TCGA GDC data portal contained information regarding somatic mutation for BRCA samples in the “MAF” format. The “Maftools” program in R software was then used to generate waterfall plots, which allowed for the visualization and summarization of all mutated genes.

### Survival analysis

For comparing the OS values between the high- and low-ICD risk cohorts, Kaplan-Meier (KM) analysis was carried out with the survminer and survival package in R software. Univariate Cox regression analysis was used to generate prospective prognostic markers, and the multivariate Cox regression analysis was employed for determining if the risk score could be used as a risk factor for OS in BRCA in an independent manner.

### Designing the ICD-Linked risk signature

In this study, the survival status, survival time, and gene expression data were integrated with the R package of glmnet. The Lasso-Cox technique was used for carrying out regression analysis. To construct the best model, 10-fold cross-validation was also performed.

### Tumor IMmune estimation resource

The researchers used the Tumor IMmune estimation resource (TIMER) algorithm dataset (https://cistrome.shinyapps.io/timer/) to investigate the correlation between the ICD-associated mRNAs expression level in BRCA. The TIMER dataset includes information regarding 32 different cancer types and contains 10,897 samples derived from The Cancer Genome Atlas (TCGA). It also includes data related to the immune cells like CD8^+^ T cells, CD4^+^ T cells, macrophages, neutrophils, B cells, and dendritic cells.

### Connectivity map database

Connectivity map (CMap) (https://clue.io) applies a systematic approach to reveal interactions among drugs, compounds, and diseases based on alterations in the genetic backgrounds of BRCA patients. Drugs potentially related to BRCA were found by uploading up-regulated genes and down-regulated genes in ICD-associated genes.

### Statistical analyses

To perform statistical analysis, the R language (version 4.0.1) was used. KM survival analysis was performed along with a log-rank test to compare survival. *p* < 0.05 was considered to indicate a statistically significant difference.

## Results

### Use of consensus clustering technique for identifying 2 ICD-Associated subtypes

**FIGURE 1:**
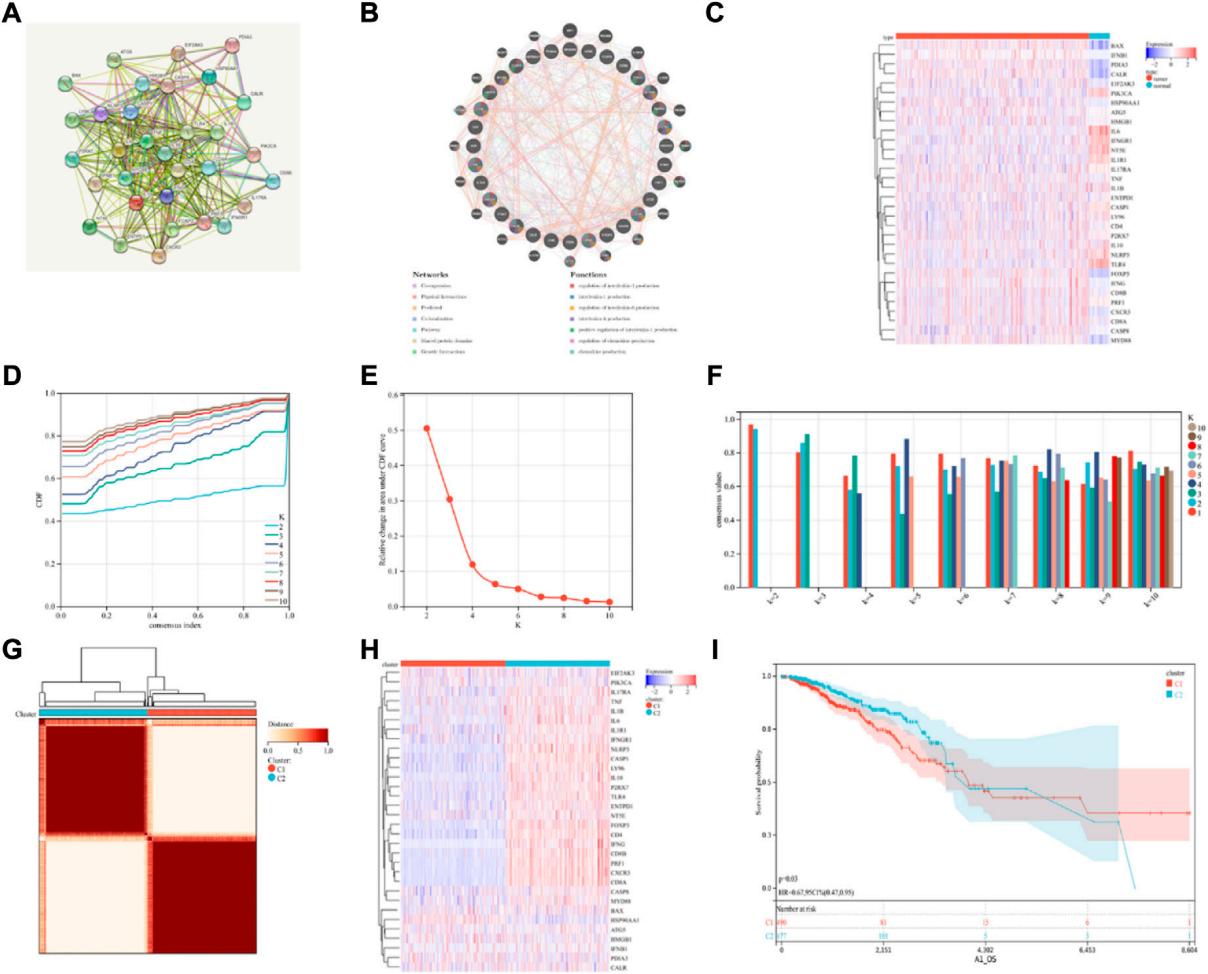
Consensus clustering identification of ICD-related subgroups. **(A)** Protein-protein interactions among the genes related to ICD using STRING; **(B)** Protein-protein interactions among the genes related to ICD using GeneMANIA; **(C)** Heatmap displaying the expression of 32 ICD genes in the BRCA and normal tissue samples using the TCGA database; **(D–F)** The consensus clustering delta area curve shows the relative difference (variations) in the area under cumulative distribution function (CDF) curve when k = 2 to 10; **(G)** The consensus clustering solution (k = 2) for 32 genes in 993 BRCA samples is shown on the heatmap; **(H)** The expression profiles of 32 ICD-related genes are shown on the heatmap. **(I)** KM curves of the patient’s OS in the ICD-low and ICD-high subcategories, where red indicated a higher expression and blue indicates a lower expression level.

Abhishek et al. (Garg et al., 2016) presented a cohort of 34 ICD-associated genes. Here, a total of 32 ICD-associated genes were detected, which were expressed in BRCA, using the TCGA database. Thereafter, the PPI network was analyzed with the STRING database for determining the relationships between the identified ICD-associated genes (Figure 1A). The GeneMANIA data also showed that the interactors and genes linked to ICD that were differently expressed were primarily involved in controlling the production of interleukin-6, etc. (Figure 1B). ICD gene expression varied substantially between normal and the BRCA samples (Figure 1C). Then we determined the predictive value of the DNA methylation status of the ICD-associated genes exclude PDIA3, IFNB1, CXCR3 in BRCA using MethSurv (Supplementary Figures S1, S2). DNA methylation expression levels concluded that cg10858077 of FOXP3, cg21721489 of HMGB1, cg13263472 of HSP90AA1, cg11354472 of IL17RA, cg01351089 of MYD88, cg12121075 of P2RX7, cg26971585 of PRF1 and cg09637172 of TNF had the highest DNA methylation levels and significant prognostic value (likelihood ratio (LR) test *p*-value < 0.05) in BRCA (Supplementary Table S1).

Then, using consensus clustering, two ICD-linked BRCA clusters were identified. Two clusters, determined by the TCGA dataset, were found to have distinctive ICD gene expression profiles after the K-means clustering analysis (Figures 1D–G). Low expression of ICD-linked genes in cluster C1 denoted an ICD-low subcategory. Cluster C2, on the other hand, displayed an overexpression, which indicated the ICD-high subcategory (Figure 1H). The C1 cluster included the ICD-low category, whereas the ICD-high subgroup was designated as cluster C2. Additionally, survival analysis revealed that the clinical outcomes of these ICD-based subcategories varied. Compared to the ICD-high subcategory, the ICD-low group was linked with lower survival (Figure 1I).

### Identifying the DEGs and signal pathways in the various ICD subcategories

**FIGURE 2:**
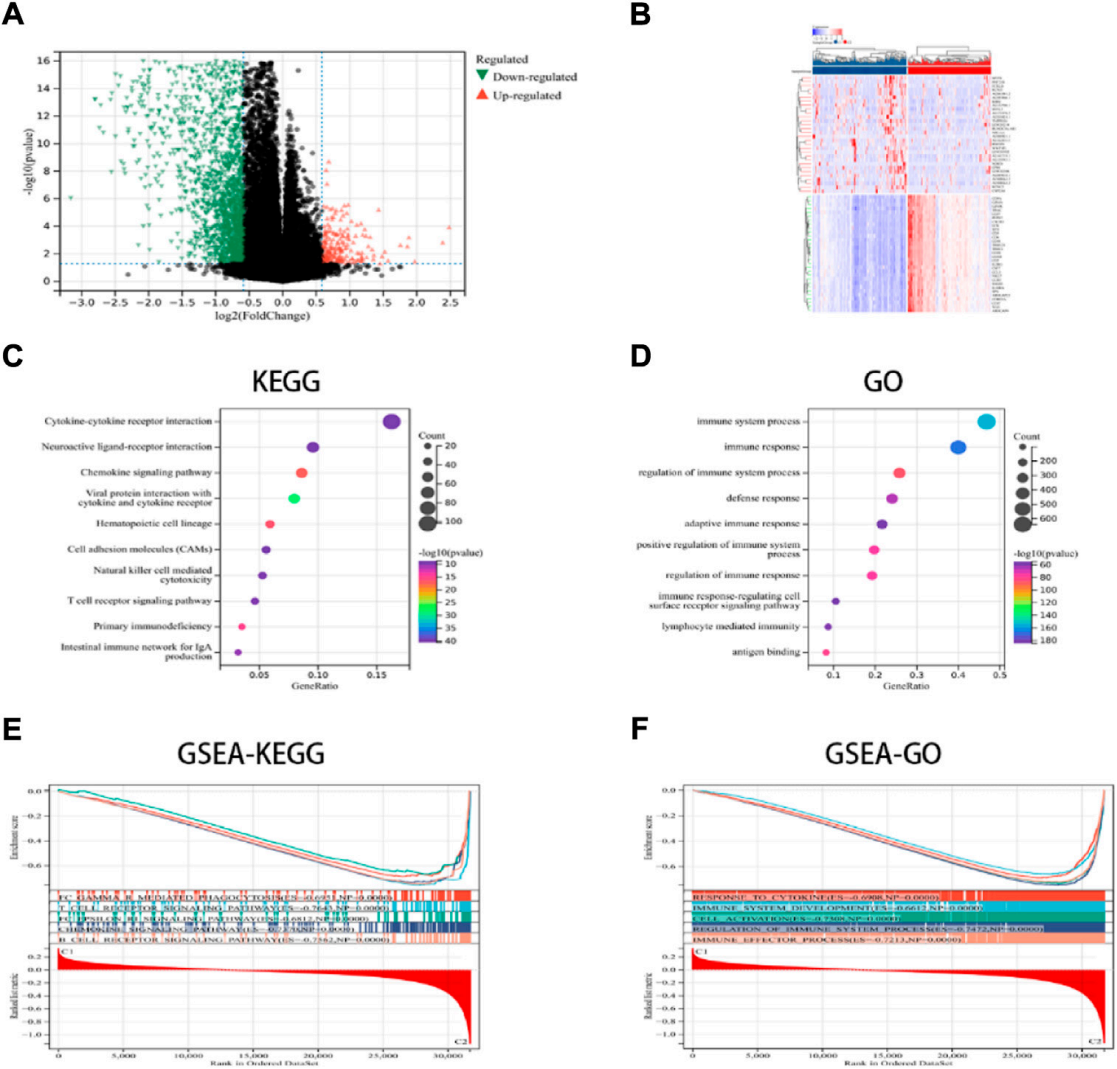
DEGs and associated signaling pathways are identified. **(A)** The distribution of the DEGs between the ICD-low and ICD-high subcategories in the TCGA dataset; **(B)** The expression of DEGs in various subgroups; and **(C,D)** the GO and KEGG enrichment analyses of signaling pathways; **(E,F)** The GSEA identifies the fundamental signal pathways present in both the ICD subcategories.

In this study, the key DEGs and vital signaling pathways involved in both the categories were identified for understanding the molecular mechanisms modulating prognosis in the two ICD subcategories. A total of 2799 dysregulated genes were detected (Figures 2A,B), and these genes were enriched in immune system functions such as cytokine-cytokine receptor interaction, immune responses, adaptive immune response, chemokine signaling pathway, NK-cell mediated cytotoxicity, Th1 and Th2 cell differentiation, etc. (Figures 2C,D). These findings implied that the ICD-linked genes were related to the immunological microenvironment. The GSEA technique was used for comparing both the ICD-low and ICD-high subcategories, thus further determining the crucial signal transduction processes that were induced in the ICD-high category. Furthermore, immunological system development, T cell receptor signaling pathways, immune effector processes, and B cell receptor signaling pathways all showed differential enrichment (Figures 2E,F).

### Somatic mutations and the tumor microenvironment in different subtypes

**FIGURE 3:**
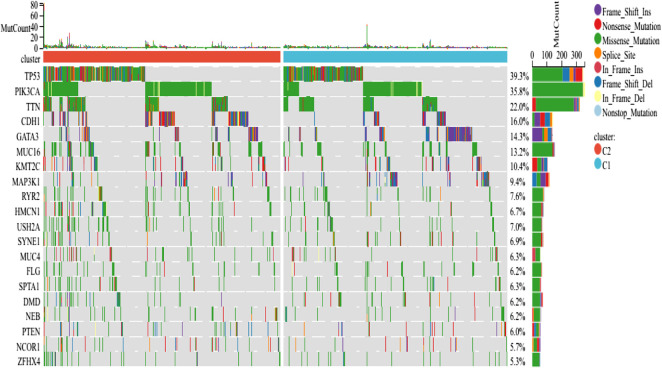
Comparing the somatic mutations occurring in the various ICD subgroups.

**FIGURE 4:**
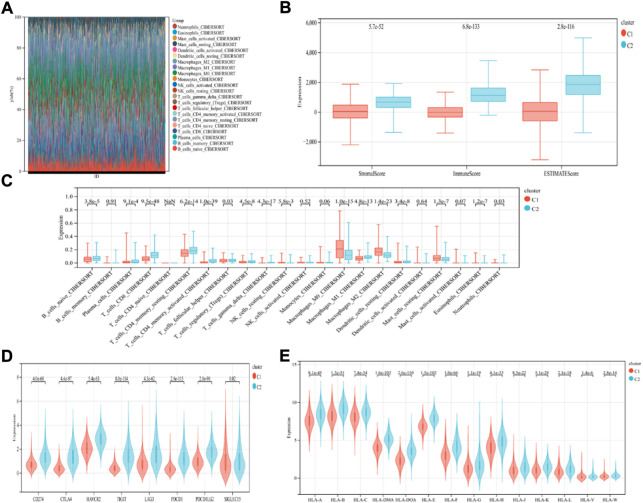
Immunological profiles of the different ICD subcategories. **(A)** Relative Proportion of the infiltration of immune cells in the ICD-low and ICD-high subcategories; **(B)** Stromal, Immune, and Estimate scores in the ICD-low and ICD-high subcategories; **(C)** The significant variations in the infiltration of immune cells in both subcategories; **(D,E)** The differential expression of numerous immune checkpoint genes **(D)**; and the HLA genes **(E)**, in the 2 subtypes.

The findings revealed that distinct somatic mutation profiles between the two subtypes (Figure 3). The most frequently mutated genes were TP53, PIK3CA, TTN, and CDH1. Besides, the mutation frequency was higher in the ICD-high subcategory in comparison to the ICD-low subcategory. There have been amounting evidences that ICD can affect the induction of specific antitumor immune responses. Here, the differences in the Tumor Microenvironment (TME) were also determined between the 2 subtypes. Figure 4A depicted the immune infiltration state of 993 BRCA patients, from the TCGA in its entirety. In comparison to the ICD-low subcategory, the ICD-high subtype showed higher immune, Stromal, and Estimate scores (Figure 4B). Then, the immunological infiltration levels of the 22 immune cells between both the subtypes were determined using the Cibersort strategy as well as the lm22 feature matrix. In comparison to patients within an ICD-high subcategory, those with the ICD-low subcategory showed considerably reduced levels of activated CD4^+^T cell memory, B-cell plasma, CD8^+^ T cells, T-cell regulation, resting CD4^+^ T cell memory, macrophages M1 and M2, eosinophils, and activated myeloid dendritic cells (Figure 4C). Additionally, the ICD-high subcategory showed a higher expression of several Human Leukocyte Antigen (HLA) genes and immunological checkpoints than the ICD-low subcategory (Figures 4D,E).

### Development and verification of the ICD risk signature

**FIGURE 5:**
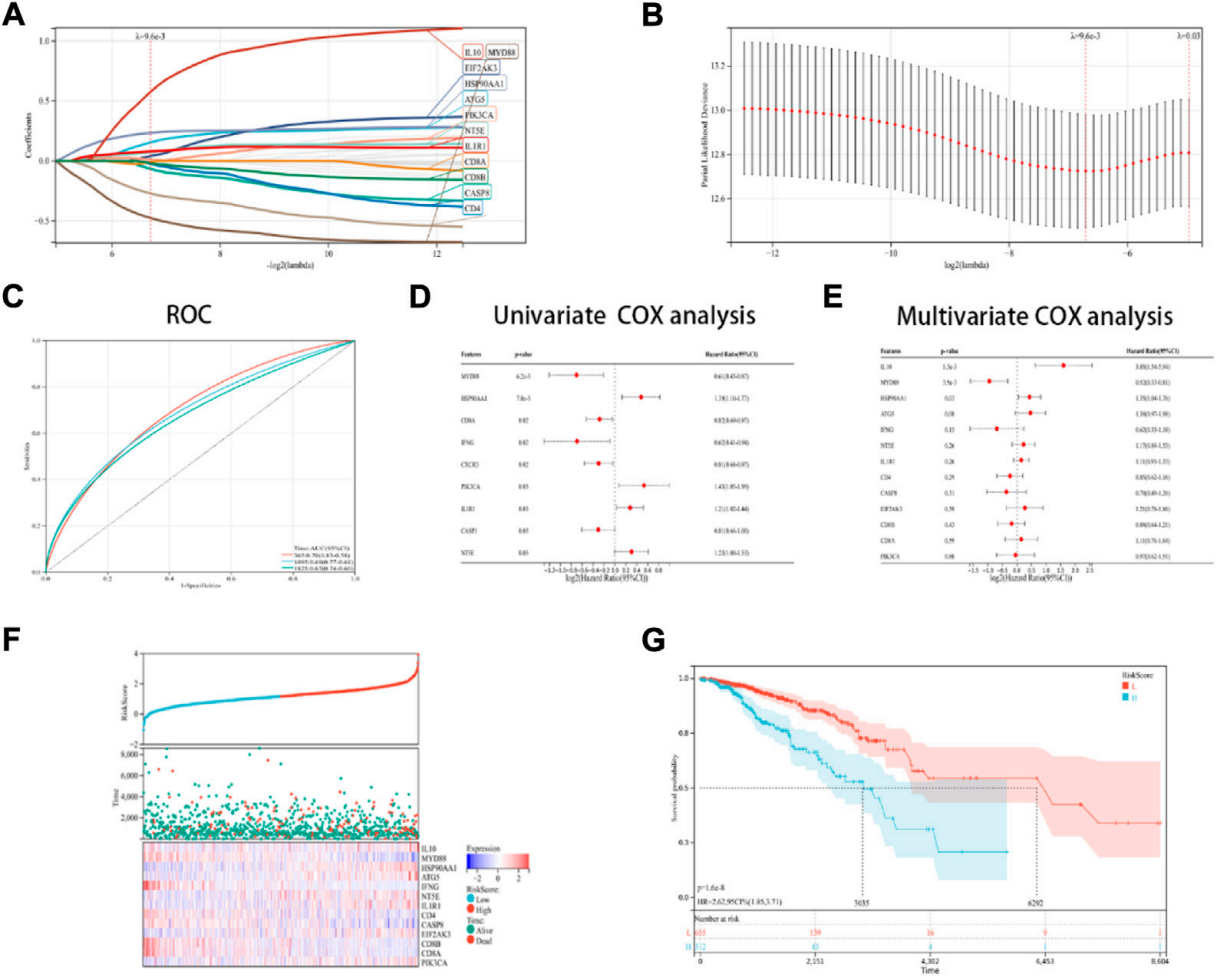
Designing and validating the ICD risk signature. **(A,B)** Lasso Cox regression analysis was used to identify 13 genes in the TCGA dataset that were strongly associated with OS; **(C)** ROC curves with the AUC values; **(D,E)** Univariate and multivariate Cox regression analyses were performed for assessing the prognostic values of the ICD genes, with regards to OS; **(F)** Risk score distribution, OS status of every patient, and the heatmaps depicting the prognostic application of the 13-gene signature; and **(G)** KM curves of the patient’s OS in the high-risk cohort and low-risk cohort subcategories.

In this research, a novel prognosis model was developed based on the ICD-linked genes. The Lasso regression analysis was used for evaluating 13 ICD-linked genes and selected them as predictive models (Figures 5A,B). With the lambda value set to 0.009563, 13 genes were identified. The following model formula was used: Riskscore = 0.1441*ATG5-0.0659*CASP8+0.0399*EIF2AK3+0.0112*PIK3CA+0.2334*HSP90AA1+0.0725*NT5E+0.0787*IL1R1-0.4761*MYD88–0.2712*IFNG+0.5758*IL10–0.0241*IL10–0.0241*CD4-0.0094*CD8A-0.0186*CD8B.

With AUC values set at 0.70 for a 1-year period, 0.79 for a 3-year period, and 0.67 for a 5-year period, the ROC curves were depicted (Figure 5C). Then, it was noted that 9 ICD-linked genes (Figure 5D) and 3 ICD-linked genes (Figure 5E) were significantly correlated with the OS values of the ICD patients using the Cox univariate regression analysis and multivariate analysis, respectively. In addition, the correlation between the riskscores and OS status was also assessed. The results indicated that fewer patients in the high-risk dataset survived (Figure 5F), with fewer surviving in the high-risk cohort (Figure 5F). KM analysis was used for determining the short OS status of the ICD patients in the high-risk cohort (Figure 5G).

## The relationship between the ICD risk signatures and the TME

**FIGURE 6:**
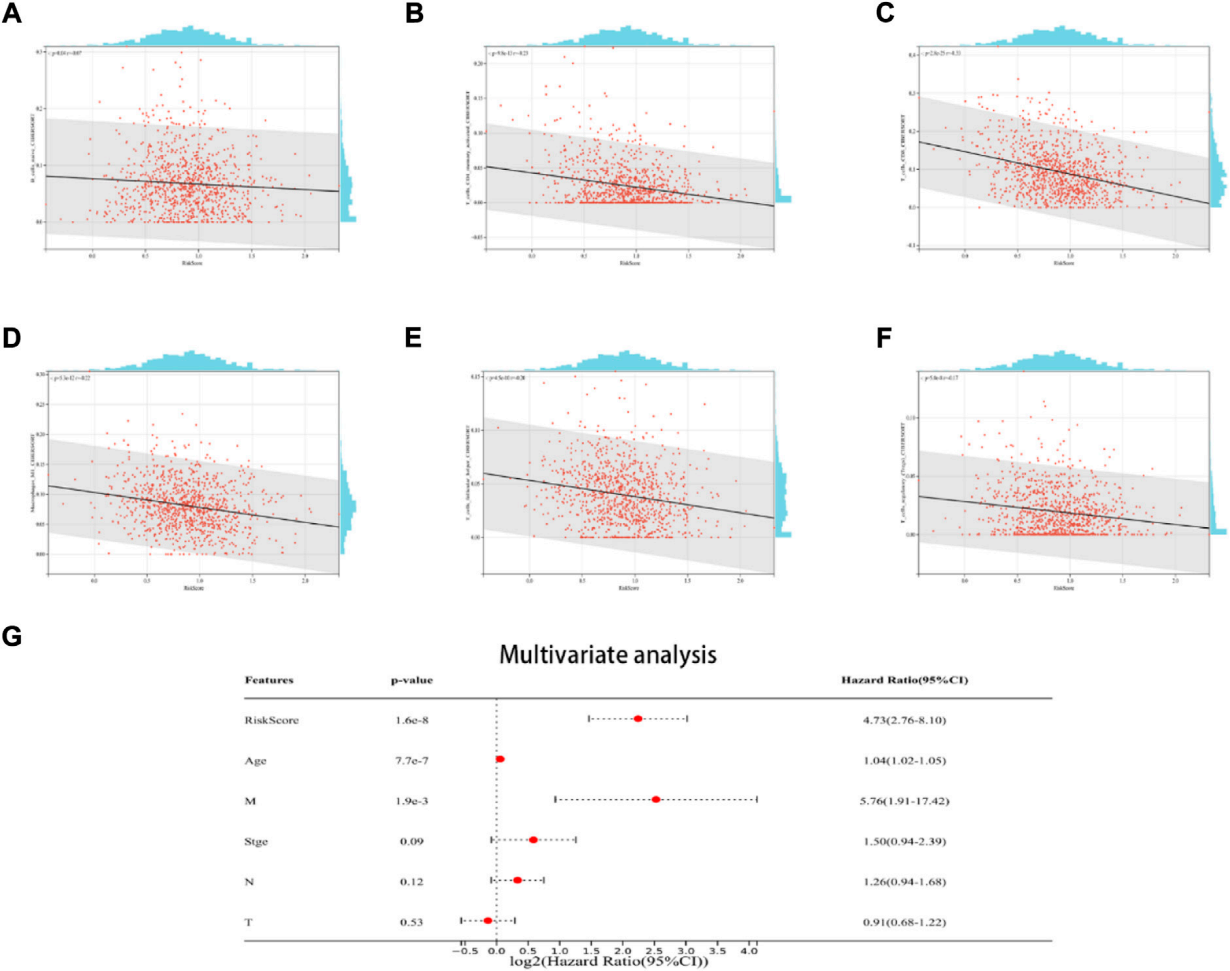
The relationship between the TME and ICD risk signature. Scatter plots in **(A–F)** illustrate the relationship between risk score and the infiltration status of activated CD4 memory cells, native B cells, CD8 cells, helper follicular T cells, M1 macrophages, and regulatory (Tregs) T cells; **(G)**: Multivariate Cox regression analysis assess the prognostic value of the ICD risk signature in BRCA patients in an independent manner.

The findings demonstrated a negative link between native B cells, active CD4 memory cells, M1 macrophages, helper follicular T cells, CD8 cells, and regulatory (Tregs) T cells among individuals with elevated risk scores (Figures 6A–F). Additionally, the multivariate Cox regression analysis revealed that the ICD risk score can be utilized as a potential prognostic factor for BRCA patients in an independent manner (Figure 6G).

### Correlation analysis between ICD-associated mRNAs expression level and the infiltrating immune cells

**FIGURE 7:**
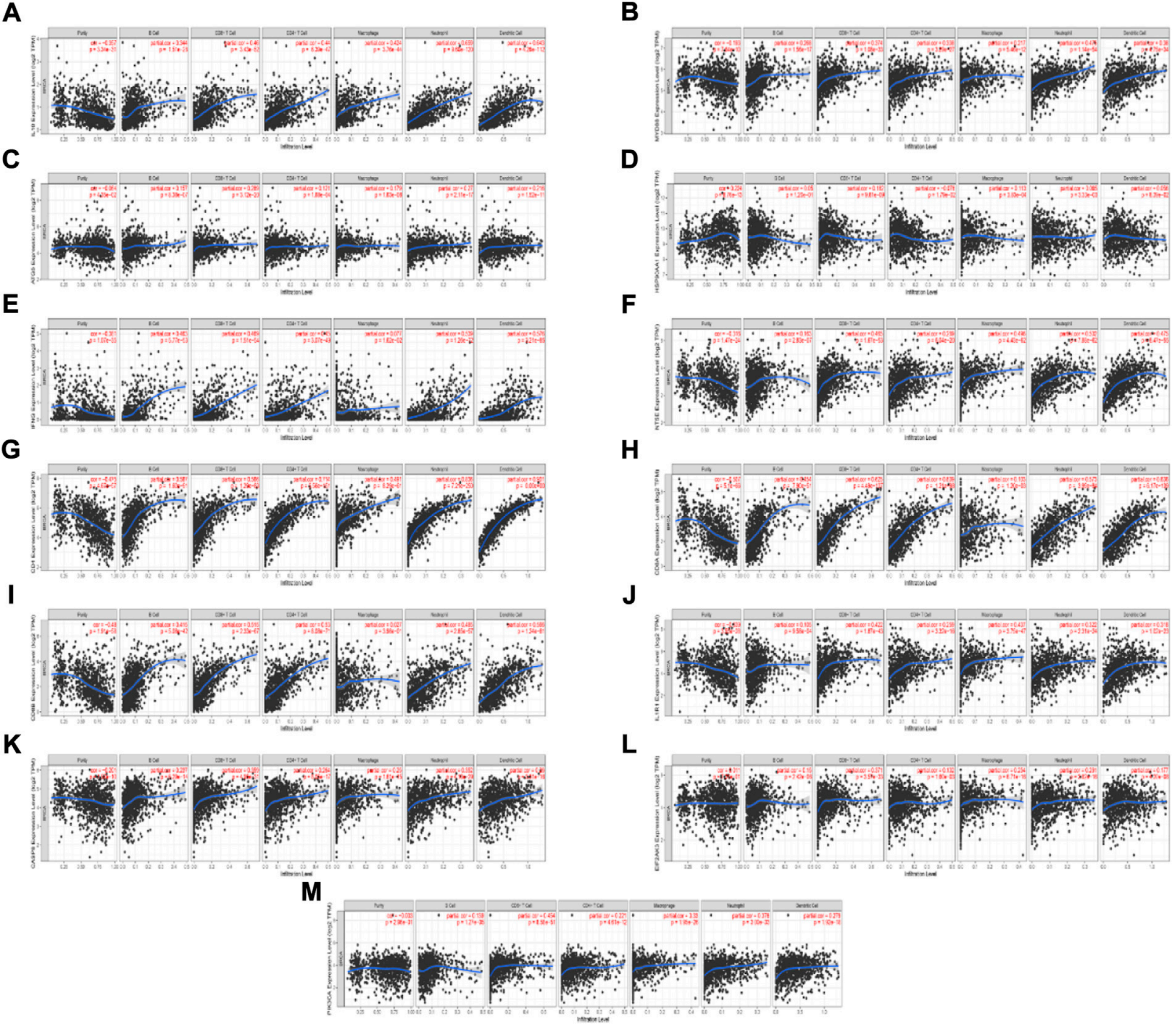
Relationship between the ICD-associated mRNAs expression levels and the immune cell infiltration level using TIMER. **(A–M)** IL10, MYD88, ATG5, HSP90AA1, IFNG, NT5E, CD4, CD8A, CD8B, IL1R1, CASP8, EIF2AK3, PIK3CA expression levels and the immune cell infiltration level.

We found 13 ICD-associated mRNAs by Lasso-cox analysis, and investigated the relationships between these mRNAs expressions and 6 types of infiltrating immune cells (neutrophils, macrophages, B cells, dendritic cells, CD4 T cells, and CD8^+^ T cells) with the aid of the TIMER database. The results are presented in Figure 7. The results showed that the 13 ICD-associated mRNAs expressions were positively correlated with the infiltration of 6 types of immune cells.

### Identification of drugs related to ICD-associated mRNAs

From the most significantly beneficial drugs, we identified 10 positively correlated drugs by CMAP, include pirenperone, lidocaine, venlafaxine, tocainide, sulfasalazine, corynanthine, candesartan-cilexetil, fomocaine, phenylbutazone and lorazepam (Table 1).

**TABLE 1:** Summary of predicted CMap drugs.

Drug name	Pharmacologic action	Average mean score
pirenperone	Serotonin receptor antagonist	0.34
lidocaine	Histamine receptor agonist	0.27
venlafaxine	Serotonin receptor antagonist|Adrenergic receptor antagonist|Norepinephrine reuptake inhibitor	0.44
tocainide	Sodium channel inhibitor	0.45
sulfasalazine	Antirheumatic drug|NFKB inhibitor	0.35
corynanthine	Adrenergic receptor antagonist	0.30
candesartan-cilexetil	Angiotensin receptor antagonist	0.32
fomocaine	Voltage-gated sodium channel blocker	0.32
phenylbutazone	Cyclooxygenase inhibitor|Prostanoid receptor antagonist	0.31
lorazepam	Benzodiazepine receptor agonist	0.34

## Discussion

ICD is a distinct type of controlled cell death. As a form of RCD, ICD orchestrates complex intercellular communication between dying cancer cells and immune cells, which then triggers antitumor innate and adaptive immunity (Krysko et al., 2012; Riera Romo, 2021; Ye et al., 2021). There are 2 types of ICD-induced mechanisms (Asadzadeh et al., 2020). The principal consequences of endoplasmic reticulum (ER) stress cause type I ICD to elicit ICD-associated danger signals. ICD-related immunogenicity is caused by type II ICD, ER, and cell death signals. Anticancer drug-induced ICD is a Type I form of ICD that functions by inducing autophagy *via* the unfolded protein response (UPR)-ER stress pathway. One aspect of cancer therapy is ICD-based induction, which involves killing the cancer cells so that it can generate a long-lasting antitumor immunity similar to the body’s natural antitumor-based immune response. It is now unclear if ICD induced by anticancer medications can enhance the prognosis of patients with BRCA by supplying long-term antitumor immune memory. Preclinical investigations have demonstrated that the ICD triggers tumor-related immune responses *via* CTLs, which act as long-term immunological memory and prevent distant relapse. However, there is inadequate clinical evidence to confirm and validate this hypothesis.

In this context, it is important to identify the immunological components needed to achieve long-lasting antitumor immunity. A combination therapy incorporating anticancer drugs and vaccines or ICIS, in addition to different anticancer drugs in development, could improve antitumor immunity by regulating immunity. Identification of biomarkers linked to ICD would therefore be advantageous for BRCA, assuming that they could benefit from immunotherapy. Herein, the findings revealed that the TME and prognosis of BRCA were highly correlated with the expression of genes linked to ICD. By using the consensus clustering technique based on ICD-linked gene expression, two ICD subtypes were identified. A positive clinical result and higher immune cell infiltration were linked to the ICD-high subcategory. In addition, using 13 chosen ICD-related genes, a prognostic risk signature was designed and validated, thus classifying the BRCA patients into the low-risk and high-risk cohorts. Additionally, this risk profile has a good prognostic value for OS status and might be used independently in predicting the outcomes of BRCA patients.

Abhishek et al. (Garg et al., 2016) presented a cohort of the ICD-associated genes, which were used in this study. Three of the 32 ICD-linked genes identified in this study had a significant impact on the prognosis of BRCA patients. The TME, composed of stromal cells (myoepithelial cells, fibroblasts, vascular endothelial cells), immune cells (including T cells, B cells, macrophages and natural killer cells) and the extracellular matrix, is a major regulator of carcinogenesis, tumour progression and response to therapy. The TME in BRCA is highly heterogeneous (Wilson et al., 2022). The TME is modified by cancer therapy-induced ICD (Zhou et al., 2019; Tu et al., 2020; Yang et al., 2020). By releasing antigens and adjuvant factors, ICD expresses its immunoregulatory capacity and modulates the tumor microenvironment (TME) through “cold-warm-hot” immune status, thereby improving T-cell priming and ultimately facilitating T-cell-mediated attack of residual killer cancer cells (CCS) (Day et al., 2021; Wang et al., 2021). ICD occurs simultaneously with the exposure and production of a variety of DAMPs, which promotes their interaction with the cognate PRRS exhibited by the innate immune cells like DCs, macrophages, and monocytes. As a result, these cells get activated and matured and travel to the draining lymph nodes that are filled with the cancer-based antigen-related payloads. When T cells are exposed to cancer antigens, the infiltration degree of immune cells into the TME is elevated (Serrano-Del Valle et al., 2019; Sansone et al., 2021). Based on this scenario, this study has identified 2 ICD subtypes using consensus clustering. To validate the relationship between ICD-associated genes and TME, we used the timer database and found that the expression of ICD-associated genes exhibited a positive correlation with infiltrating immune cells.

Currently, many studies have shown that tumor immunogenicity can be increased by triggering specific cell death forms in cancer cells. For example, chemotherapy (Casares et al., 2005), radiotherapy (Krombach et al., 2018) and photodynamic therapy (PDT) (Morais et al., 2021; Rodrigues et al., 2022a), can induce immunogenic cell death (ICD), which can make cancers more effective in triggering or enhancing tumor antigen-specific immune responses. Current studies recommend ICD induction in combination with other immunotherapeutic strategies, such as immune checkpoint blockade, to be explored for the treatment of cancer. However, barriers such as the lack of standardization of ICD testing in clinical patients and the need for more personalized protocols based on detailed characterization of tumor cells must be overcome before experimental protocols can be translated to the clinic (Rodrigues et al., 2022b). Finally, we searched for potential drugs using CMAP database to provide prospective guidance for future clinical treatment.

In conclusion, this research reveals that ICD subtypes are related to variations in the BRCA immunological TME. These findings could help BRCA patients who are undergoing immunotherapy-based treatments. Furthermore, an ICD-linked prognostic signature model was developed and verified, which could be vitally useful in predicting OS of the BRCA patients. These results would definitely guide future clinical interventions to a certain extent. Undeniably, we still have limitations, as exemplified by the adoptionof publicly available databases, and further experiments are needed to validate the conclusions of this study.

## Data Availability

The datasets presented in this study can be found in online repositories. The names of the repository/repositories and accession numbers can be found in the article/Supplementary Material.
