# Supplementary material for: An immunogenic cell death-associated classification predictions are important for breast invasive carcinoma prognosis and immunotherapy
Source: Front Genet. 2022 Oct 20;13:1010787. doi: 10.3389/fgene.2022.1010787 (PMC9630734; doi:10.3389/fgene.2022.1010787)
Supplement: Supplementary file 1 [file DataSheet1.PDF]

Supplementary Materials: Figure S1: Heatmap of DNA methylation expression levels of the ICD-associated genes in breast cancer by MethSurv platform. (A-O) cg04389950 of ATG5; cg22237988 of BAX; cg13466180 of CALR; cg13802966 of CASP1; cg14930754 of CASP8; cg15409796 of CD4; cg00219921 of CD8A; cg08777095 of CD8B; cg05707116 of EIF2AK3; cg18054998 of ENTPD1; cg10858077 of FOXP3; cg21721489 of HMGB1; cg13263472 of HSP90AA1; cg01940810 of IFNG; cg02480602 of IFNGR1 displays the highest level of DNA methylation in BRCA.

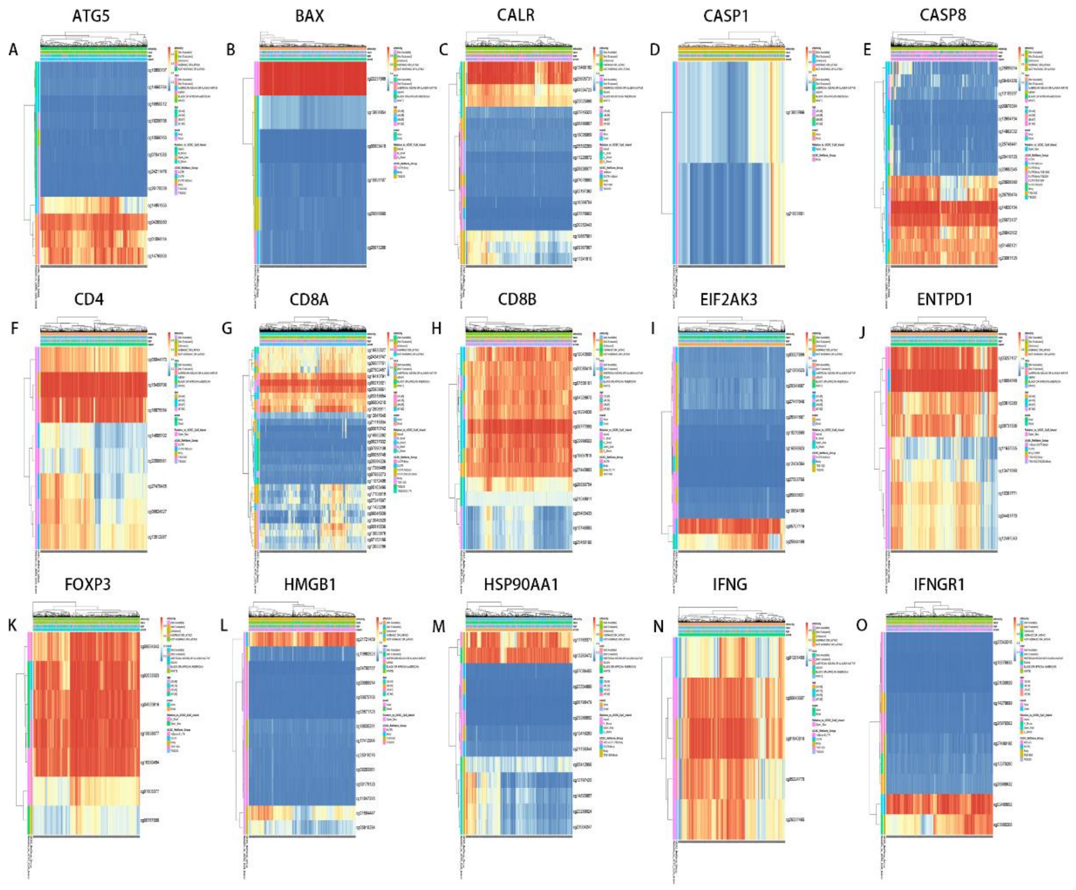

Figure S2: Heatmap of DNA methylation expression levels of the ICD-associated genes in breast cancer by MethSurv platform. (A-N) cg18635064 of IL1B; cg15233681 of IL1R1; cg02335517 of IL6; cg17067005 of IL10; cg11354472 of IL17RA; cg17503786 of LY96; cg01351089 of MYD88; cg18793688 of NLRP3; cg24702826 of NT5E; cg12121075 of P2RX7; cg13154908 of PIK3CA; cg26971585 of PRF1; cg14629571 of TLR4; cg09637172 of TNF displays the highest level of DNA methylation in BRCA.

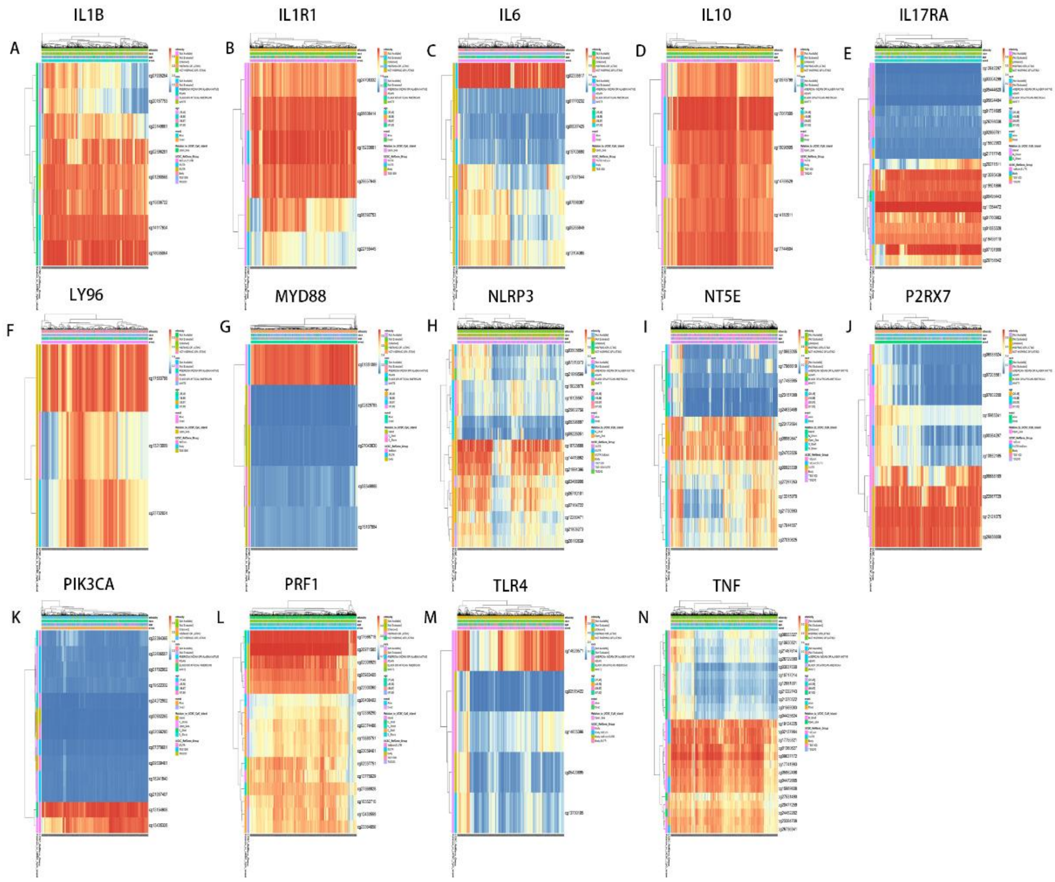

Table S1: Prognostic Value of Single CpG of the ICD-associated genes in BRCA by MethSurv platform. The threshold of significance was LR Test p-value. A significant expression pattern was found in FOXP3, HMGB1, HSP90AA1, IL17RA, MYD88, P2RX7, PRF1 and TNF between low and high risk groups for BRCA. (* $p < 0.05$)

Gene-CpG	HR	LR_test_pvalue
ATG5-cg04389950	1.353	0.21
BAX-cg22237988	0.763	0.17
CALR-cg13466180	0.823	0.33
CASP1-cg13802966	0.824	0.42
CASP8-cg14930754	0.715	0.14
CD4-cg15409796	1.635	0.056
CD8A-cg00219921	1.336	0.17
CD8B-cg08777095	1.452	0.099
EIF2AK3-cg05707116	0.881	0.57
ENTPD1-cg18054998	0.818	0.31
FOXP3-cg10858077	1.867	0.005*
HMGB1-cg21721489	0.611	0.014*
HSP90AA1-cg13263472	0.651	0.042*
IFNG-cg01940810	1.335	0.19
IFNGR1-cg02480602	0.683	0.12
IL1B-cg18635064	1.239	0.32
IL1R1-cg15233681	1.38	0.18
IL6-cg02335517	1.426	0.15
IL10-cg17067005	0.887	0.61
IL17RA-cg11354472	0.524	0.0068*
LY96-cg17503786	1.082	0.69
MYD88-cg01351089	0.621	0.043*
NLRP3-cg18793688	1.21	0.42
NT5E-cg24702826	0.67	0.1
P2RX7-cg12121075	0.631	0.024*
PIK3CA-cg13154908	0.723	0.19
PRF1-cg26971585	1.791	0.0039*
TLR4-cg14629571	1.206	0.43
TNF-cg09637172	1.726	0.027*
